# Serological Response of Patients with Influenza A (H1N1) pdm09-Associated Pneumonia: An Observational Study

**DOI:** 10.1371/journal.pone.0081436

**Published:** 2013-11-27

**Authors:** Nasikarn Angkasekwinai, Bualan Kaewnapha, Duangdao Waywa, Peerawong Werarak, Sasima Tongsai, Kulkanya Chokephaibulkit, Visanu Thamlikitkul, Sontana Siritantikorn

**Affiliations:** 1 Department of Medicine, Faculty of Medicine Siriraj Hospital, Mahidol University, Bangkok, Thailand; 2 Department of Microbiology, Faculty of Medicine Siriraj Hospital, Mahidol University, Bangkok, Thailand; 3 Department of Pediatrics, Faculty of Medicine Siriraj Hospital, Mahidol University, Bangkok, Thailand; University of Oxford, Viet Nam

## Abstract

**Background:**

Little is known about the dynamics or magnitude of antibody response in patients with influenza A (H1N1) pdm09-associated pneumonia. We described and compared the antibody response to influenza A (H1N1) pdm09 in patients with and without pneumonia.

**Methods:**

We collected serum samples and determined antibody titers by the hemagglutination inhibition (HI) and microneutralization (mNT) assays from patients with RT-PCR confirmed influenza A (H1N1) pdm09 virus at baseline, 1, 2 and 6 months after onset of illness.

**Results:**

Fifty-nine patients were enrolled, 45 (76.3%) were between 15 and 60 years of age, 49 (83.1%) were hospitalized and 25 (42.4%) had complications with pneumonia. Ninety-four percent of patients had HI titers ≥ 1: 40 and 90% had mNT titers ≥ 1: 160 at 2 months after illness. Geometric mean titers (GMT) of HI and mNT increased significantly (*p*<0.001) between baseline and months 1 or 2, then declined significantly (*p*<0.001) at month 6 by the HI assay, but dropped to an insignificant level (*p*=0.24) by the mNT assay. The mNT-GMT was at least twice as high as corresponding HI antibodies over a 6 month period. The GMT of HI and mNT in those with pneumonia (1 mo) peaked earlier than that of those without pneumonia (2 mo). When adjusted by age and gender, those with pneumonia had a higher HI-GMT than those without pneumonia at 1 month (264 vs. 117, *p*=0.007), 2 months (212 vs. 159, *p*=0.013), and 6 months (160 vs. 82, *p*=0.018).

**Conclusions:**

The patients recovered from influenza A (H1N1) pdm09-associated pneumonia, clearly developed an earlier and more robust antibody response until 6 months after onset of illness. The results in our study are useful to determine an appropriate donor and timing to obtain convalescent plasma for adjunctive treatment of seriously ill patients with pandemic H1N1 influenza.

## Introduction

The pandemic A (H1N1) 2009 virus has become a seasonal virus, continuing to circulate with other seasonal virus since August 2010 when WHO declared that the H1N1 influenza virus had moved into the post-pandemic period [1]. Compared to patients with seasonal influenza, those with the influenza A (H1N1) pdm09 are younger and less likely to have underlying disease [2]. In addition to advanced age and underlying medical conditions, pregnancy and obesity were found to be associated with severe disease [3]. 

Innate and cellular immune responses play a significant role of defense against the invading influenza virus. Regarding humoral response, the antibodies, produced locally and systemically to both influenza glycoproteins, haemagglutinin (HA) and neuraminidase (NA) help to prevent the viral spread and eradicate the virus. While the hemagglutination inhibition (HI) assay detects antibodies that bind to the receptor binding site of the viral HA, blocking the interaction of HA with sialic acid receptors on erythrocyte and inhibiting their agglutination, the microneutralization (mNT) assay detects antibodies that neutralize the virus by inhibiting viral entry of HA, including antibodies recognizing epitopes within the stem region and that are conserved among viruses of different influenza A virus subtypes [4]. Therefore, the HI assay was found to be more specific whereas the neutralization test (NT) was more sensitive for detecting antibody to influenza A (H1N1) pdm09 [5]. Even though HI titers of ≥1: 40 are associated with a 50% or greater reduction in the risk of seasonal influenza infection in susceptible populations, such protective titer for the neutralization assay is not known. A previous study of antibody responses in individuals infected with pandemic influenza showed that the NT titer was generally higher than the HI titer [5]. In pediatric populations, it was found that an HI titer of 40 corresponded to an NT titer of 40, whereas it corresponded to an NT titer of 160 or greater in adults [6]. 

Generally, the production of antibody appears within two weeks, peaks between 4 to 7 weeks and remains for years even without exposure [7]. However, several factors, including advancing age, being overweight or obese or receiving immunosuppressive agents may negatively affect the antibody response to influenza virus. Increases in neutralizing antibody or hemagglutinin titers were relatively lower in older participants after vaccination against pandemic H1N1 [8,9]. Increases in BMI were also found to be correlated to a 4 fold decrease in antibody titer at 12 months after vaccination [10]. In addition, current glucocorticoid use was found to be associated with a lower seroconversion rate after H1N1 vaccination [11]. Recent data also highlighted the potential importance of gender in response to influenza vaccination [12]. Nevertheless, only a few studies evaluated antibody response after naturally acquired infection. Esposito and colleagues demonstrated that healthy children evoked adequate seroprotection after naturally acquired A (H1N1) pdm09 infection and found that a greater immune response was associated with more severe disease [13] which was similar to the study conducted in adult patients [14]. However, the long term dynamics of the antibody response in patients with influenza A (H1N1) pdm09-associated pneumonia has not been well investigated. Given the interest of using convalescent plasma therapy in patients with severe H1N1 2009 infection or in those who carried drug resistant virus, the information on long term dynamics of antibody could be beneficial to determine appropriate donors and timing to obtain plasma for treatment of those patients in the future. The aim of this study is to determine the kinetics of the antibody response in patients with influenza A (H1N1) pdm09 infection with and without pneumonia.

## Methods

### Ethics Statement

The study was approved by the Institutional Review Board of Siriraj Hospital Faculty of Medicine, Mahidol University, and was carried out at the Department of Medicine, Pediatrics and Microbiology. Written informed consent was obtained from the patient, or a parent or legal guardian, and assent was obtained in children older than 7 years who were in the condition that can provide assents. 

### Study Design

During February to October 2010, patients with influenza A (H1N1) pdm09 infection confirmed by RT-PCR, were invited to join the study and enrolled following their informed consent. We collected demographics and clinical information such as manifestations of the illnesses and treatment as well as outcomes. Serum samples were also obtained at baseline within 7 days of illness, 1 month, 2 months and 6 months after onset of illness. Sera from patients were stored at -20 °C until testing. The antibody response was measured for each serum sample by the microneutralization (mNT) and hemagglutination inhibition (HI) assays. 

### Laboratory Methods

#### The influenza A (H1N1) pdm 09 virus

Influenza A (H1N1) pdm09 was the first isolate in the Virology Laboratory Unit, Faculty of Medicine Siriraj Hospital during the novel pandemic influenza in Thailand. The virus was propagated in MDCK cells (CCL-34 ATCC, USA) with MEM (Gibco, USA) supplemented with 2% fetal bovine serum, antibiotics and TPCK–trypsin (Sigma, USA). The virus was used for detection of influenza antibody by both the HI and mNT assays. 

#### Human sera

Positive and negative control sera were received from the Virology Laboratory Unit Faculty of Medicine Siriraj Hospital. All human sera were treated to inactivate non-specific inhibitors before use in the assay. For the HI assay, 50 µl of serum was mixed with 150 µl of receptor destroying enzyme (RDE, Denka Seiken, Japan) and incubated overnight in a water bath at 37 °C followed by heat inactivation at 56 °C for 30 minutes, then the nonspecific agglutinator was removed from serum by absorbing with goose erythrocytes for 1 hour at 4 °C. For the mNT assay, 500 µl of serum was heat inactivated at 56 °C for 30 minutes and two-fold serial dilutions were prepared.

#### Hemagglutination inhibition (HI) assay

 The needed amounts of hemagglutinin antigen of the influenza A(H1N1) pdm09 virus were titrated by hemagglutination (HA) test to obtain the HA titer used in the HI assay. The virus was serially diluted two-fold with phosphate buffered saline (PBS). The diluted virus was added in duplicate at 50 µl/well to 50 µl of 0.5% goose RBC. After incubation for 30 min at 4 °C, the hemagglutinin titer of the virus was determined as an HA unit. One HA unit was a reciprocal of the highest virus dilution that had complete hemagglutinating activity. The virus titer of 4HA was adjusted for HI assay. Serial twofold dilutions starting from 1:10 serum with 25 µl/well in duplicate in V-bottom microtiter plates, were incubated with 4 HA units/25 µl virus for 30 min at room temperature (RT). Fifty µl of freshly prepared 0.5% goose RBC was added to the well and briefly agitated before further incubation for 30 min at 4 °C. Positive control and negative control serum were included on each test plate. The HI titer was defined as the reciprocal of the highest dilution that still completely inhibited the hemagglutination reaction.

#### Microneutralization (mNT) assay

Neutralizing antibody of influenza in serum was detected by the microneutralization (mNT) assay based on ELISA. The standardized virus titer for the mNT assay was determined by testing the virus in half log dilutions in quadruplicated wells of a microtiter plate. After an 18-22 h incubation with MDCK cells, the virus infected cells were detected by ELISA. The test included four wells of control cells. The test wells with an absorbance (OD490) two times or greater than that of cell control wells were scored positive for the virus. Tissue culture with 50% the infectious dose (TCID(50)) was calculated by the Reed and Muench method. To detect the NT antibody in serum, 100TCID50/50µl virus was incubated with 50 µl of each serum dilution in duplicate for 1 h at 37 °C. The test included four wells of virus control (VC), four wells of cell control (CC), as well as two wells of each dilution of virus back-titration, positive control and negative control serum. The plate was incubated at 37 °C for 1 h before adding 100 µl of the mixture to 1.5 × 10^4^ MDCK cells. The ELISA test was performed after culture incubation for 18-22 h at 37 °C, 5% CO_2_. Cells were fixed with cold acetone and washed three times with the wash buffer (PBS, 0.05% Tween 20). Mouse monoclonal antibody against Influenza A nucleoprotein (Chemicon, USA) 50 µl which were diluted in blocking buffer (PBS, 1% bovine serum albumin, 0.1% Tween 20) were added to each well. After incubation for 1 h at RT the plate was washed four times and 50 µl/well of horseradish peroxidase-conjugated goat anti-mouse IgG (chemicon, USA) was added to each well. The plate was incubated for 1 h at RT, washed six times, and then incubated with 50 µl/well of freshly prepared O-phenylenediaminedihydrochloride (OPD) (Sigma, USA) substrate in citrate buffer (Sigma, USA). The reaction was stopped after 10 min with 50 µl of stop solution. The absorbance (OD) was measured at 490 nm by a microplate reader (Biotek-ELX-800 UV, USA). The neutralizing activity was determined with the following equation: *X* = [(average *OD* of VC) − (average *OD* of CC)]/2 + (average *OD* of CC), *X* = 50% of specific signal. All values below X values were considered as having a positive neutralization activity. The Influenza NT antibody titer was evaluated by the reciprocal of the highest dilution that showed positive neutralizing activity. 

### Statistical analysis

All statistical analyses were performed using PASW statistics 18.0 (SPSS) for Windows and Microsoft Excel 2007. We defined pneumonia based on admission chest radiography using WHO criteria including the presence of a consolidation, interstitial infiltration or pleural effusions [15]. The seroprotection rate was defined as the proportion of subjects with HI titers ≥1: 40 or with NT titer ≥1: 40 in children and ≥ 1:160 in adults age 15 and older. Seroconversion was defined as the proportion of subjects with a minimum 4-fold increase in antibody titer between the baseline and follow-up blood sample. The sample with the highest titer amongst all follow-up samples for that participant was used to assess if seroconversion occurred. 

The categorical data was presented as number (%) and continuous data was presented as mean (95% CI) or median (rank) as appropriate. The categorical data between the patients with and without pneumonia and between those that did and did not seroconvert were compared by using the Fisher exact test. Antibody level was expressed as geometric mean titer (GMT) which was performed with log-transformed titers. HI or NT titers below10 were arbitrarily assigned a value of 5. A repeated-measures ANOVA with post hoc test (Bonferroni method) was used to compare the GMT of HI or NT at different time periods. Kruskal Wallis test was used to compare the difference of antibody response at each time point (month 1, 2 and 6) and among the three age groups (those younger than 15, 60 or older and those between 15-59 years). 

To compare continuous data of patients with and without pneumonia and those that did and did not seroconvert, two-sample Kolmogorov-Smirnov (K-S) test was performed to test the null-hypothesis of no difference between the distribution of two groups, then Mann-Whitney U test was used to test for differences in the location between the two groups. All multiple logistic regression analyses were initially adjusted for age; and then further adjusted for sex, underlying disease, duration from illness to treatment, and duration of antiviral treatment to evaluate the difference in GMT between those with and without pneumonia. Hosmer-Lemeshow test was used to evaluate the goodness-of-fit of the logistic regression models. A *p* value of <0.05 was considered statistically significant.

## Results

**Figure 1:**
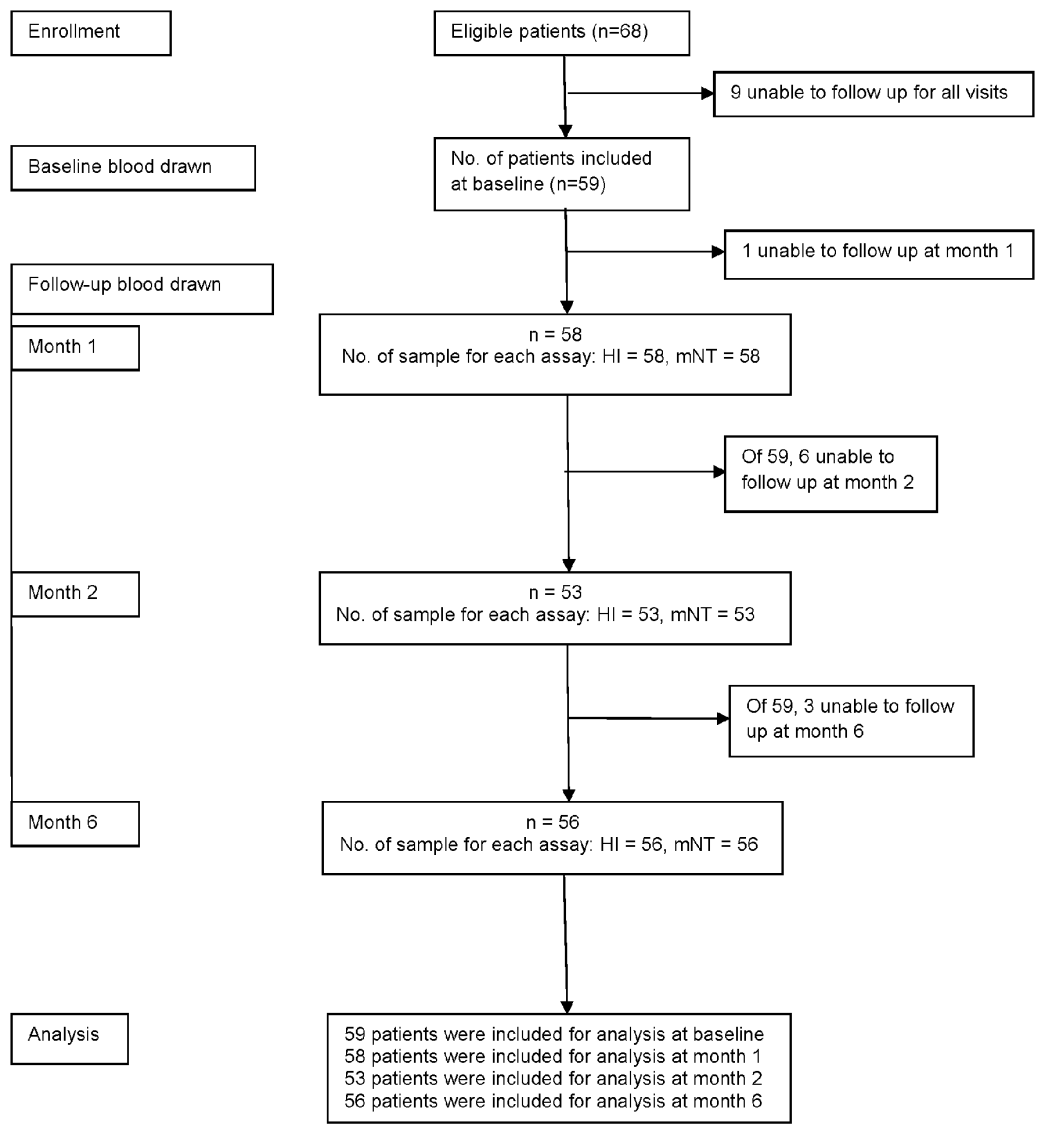
CONSORT diagram shows the flow of the study participants through 6 months period. The patients who had at least one follow-up visit remained including in the study.

Of the 68 patients with influenza A (H1N1) pdm09 who were eligible, 9 were excluded from final analysis due to lack of convalescent plasma. Fifty-nine patients had antibody titers measured at baseline, 58 at month 1, 53 at month 2 and 56 at month 6. The flow of study participants through 6 months period was shown in Figure 1. The demographics of the 59 patients were summarized in Table 1. Forty-five patients (76.3%) were between 15 and 60 years of age and only 10% were younger than 15, ranging from 5 to 13 years. Thirty-one patients (52.6%) had comorbid conditions, of which pregnancy (11.9%) was the most frequently found. Only 6 cases (10%) received the seasonal influenza vaccine. Of these, 4 received seasonal influenza vaccine without the component of influenza A (H1N1) pdm09 strain. Two cases who received trivalent inactivated influenza vaccine that contained influenza A (H1N1) pdm09 strain developed symptom at the same day or one day after getting vaccination. The majority of the patients (83.1%) were hospitalized and 25 (42.4%) had complications with pneumonia. All patients received oral oseltamivir and 8 patients also received intravenous zanamivir. The median duration from illness to treatment was 2 days (0, 7) and the median duration of treatment was 5 days (4, 10). All patients survived and were discharged after treatment. 

**Table 1:** Demographics of 59 patients infected with A (H1N1) pdm09.

Variables	n (%)
Age, years	
<15	6 (10.2)
15-29	26 (44.1)
30-44	9 (15.3)
45-59	10 (16.9)
≥60	8 (13.6)
Sex, female	38 (64.4)
Co-morbid condition	31 (52.6)
Pregnancy	7 (11.9)
Chronic heart disease	3 (5.1)
Chronic kidney disease	3 (5.1)
Diabetes	3 (5.1)
Asthma	2 (3.4)
Cancer	2 (3.4)
Obesity	2 (3.4)
Other	9 (15.2)
Seasonal influenza vaccination*	6 (10.2)
Clinical	
Fever > 37.8 c	46 (78.0)
Cough	56 (94.9)
Dyspnea	32 (54.2)
Diarrhea	13 (22.0)
Pneumonia	25 (42.4)
Hospitalization	49 (83.1)
Outcome	
Cured/ improved	59 (100)

*Of 6 cases, 2 cases received seasonal influenza vaccine containing influenza A (H1N1) pdm09 strain. The formulation of that vaccine contained A/Carifonia/7/2009 (H1N1)-like virus, A/Perth/16/2009(H3N2)-like virus and B/Brisbane/60/2008-like virus.

### Antibody response to influenza A (H1N1) pdm09 virus of all patients

**Figure 2:**
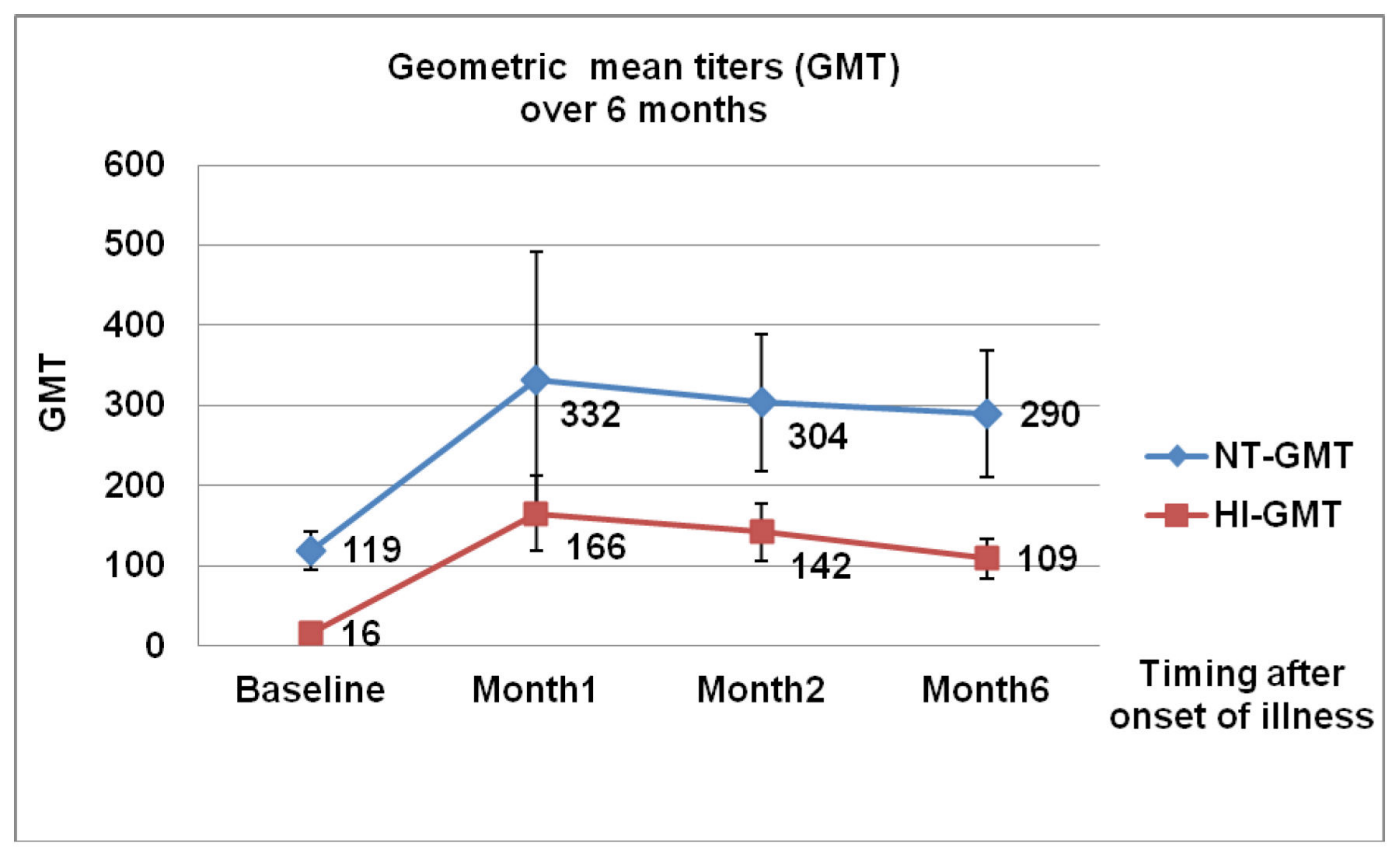
Geometric mean titers of hemagglutination inhibition (HI) and neutralization (NT) antibodies over 6 months.

The antibody response of 59 patients with A (H1N1) pdm09 infection is shown in Table 2. The raw data of all patients were made available as supplementary information (Table S1). Baseline serum samples were collected at a median of 4 days (range 1-7 days) after illness onset. Compared to baseline (16, 95% CI 13-18), the HI-GMT increased significantly (*p*< 0.001) at 1 mo (166, 95% CI 119-212) and 2 mo (142, 95% CI 107-178), then markedly declined at month 6 compared to month 2 (109 [95% CI 85-133] vs. 142 [95% CI 107-178], *p*<0.001). Likewise, there was a progressive increase of NT-GMT from baseline to month 1 and 2 but no significant decrease from month 2 to month 6 (290 [95% CI 211, 369] vs. 304 [95% CI 218, 389], *p*=0.24). The NT-GMT was at least twice as high as corresponding HI antibodies over a 6 month period (Figure 2). The seroprotection rate determined by the HI and mNT assays increased markedly at 2 month compared to baseline (HI 94.3% vs. 32%, NT 90.6% vs. 52.5%, respectively).

**Table 2:** Antibody response of 59 patients with A (H1N1) pdm09 infection.

Timing after illness onset	Hemagglutination inhibition (HI) assay			Microneutralization (mNT) assay	
	n	HI-GMT (95%CI)	Seroprotection^π^ n(%)	NT-GMT (95%CI)	Seroprotection^π^ n(%)
Baseline	59	16 (13, 18)	19 (32.0)	119 (95, 144)	31(52.5)
1 month	58	166 (119, 212)*	55 (94.8)	332 (171, 492)*	50 (86.2)
2 month	53	142 (107, 178)*	50 (94.3)	304 (218, 389)*	48 (90.6)
6 month	56	109 (85, 133)^†^	52 (92.8)	290 (211, 369)	49 (87.5)

Abbreviation: GMT=geometric mean titer; CI=confidence interval.

^π^Seroprotection rate was defined as the proportion of subjects with HI titers ≥1: 40 or with NT titer ≥1: 40 in pediatrics and ≥ 1:160 in adult age ≥ 15 years.

*p* < 0.05* was considered significant comparing baseline and months1 and 2 within the HAI or NT arm.

*p* < 0.05† was considered significant comparing months 6 and months 2 within the HAI or NT arm.

Table 3 shows demographics and characteristics of patients with and without seroconversion determined by the HI and mNT assays. The results of two-sample K-S test showed that there were no differences in distributions of data between two groups. A seroconversion rate of at least a four-fold increase in titer was observed in 81.4% (48/59) and 54.2% (32/59), determined by HI and mNT, respectively. There was no significant difference in age, gender, co-morbid condition, duration from illness to treatment, and diagnosis of pneumonia between patients who did and did not seroconvert. However, the patients who did seroconvert were more likely to have a baseline HI titer < 1:40 than those who did not seroconvert (38/48(79.2%) vs. 2/11 (18.2%), *p*<0.001). 

**Table 3:** Demographics and characteristics of patients with and without seroconversion determined by hemagglutination Inhibition (HI) and microneutralization (mNT) assays.

Variables	Hemagglutination inhibition (HI) assay			Microneutralization (mNT) assay		
	Seroconversion (n=48)	No seroconversion (n=11)	*p*-value	Seroconversion (n=32)	No seroconversion (n=27)	*p*-value
Age (years)*	29 (5,70)	28 (13,60)	0.572	31 (5,68)	28 (13,70)	0.784
Gender, female, n(%)	29 (60.4)	9 (81.8)	0.297	19 (59.4)	19 (70.4)	0.424
Co-morbid condition	26 (54.2)	5 (45.5)	0.741	16 (50)	15 (55.6)	0.795
Duration from illness to treatment (days)*	2 (0,7)	2 (0,5)	0.623	2 (0,7)	2 (0,6)	0.342
Diagnosis of pneumonia, n (%)	21 (43.8)	4 (36.4)	0.745	16 (50)	9 (33.3)	0.290
Low baseline antibody titer^@^	38 (79.2)	2 (18.2)	<0.001	19 (59.4)	9 (33.3)	0.067

*Reported as median (rank)

^@^ Baseline HI titers <1:40 or baseline mNT titers <1:40 in pediatrics and < 1: 160 in adult

### Comparison of antibody response among different age groups

**Figure 3:**
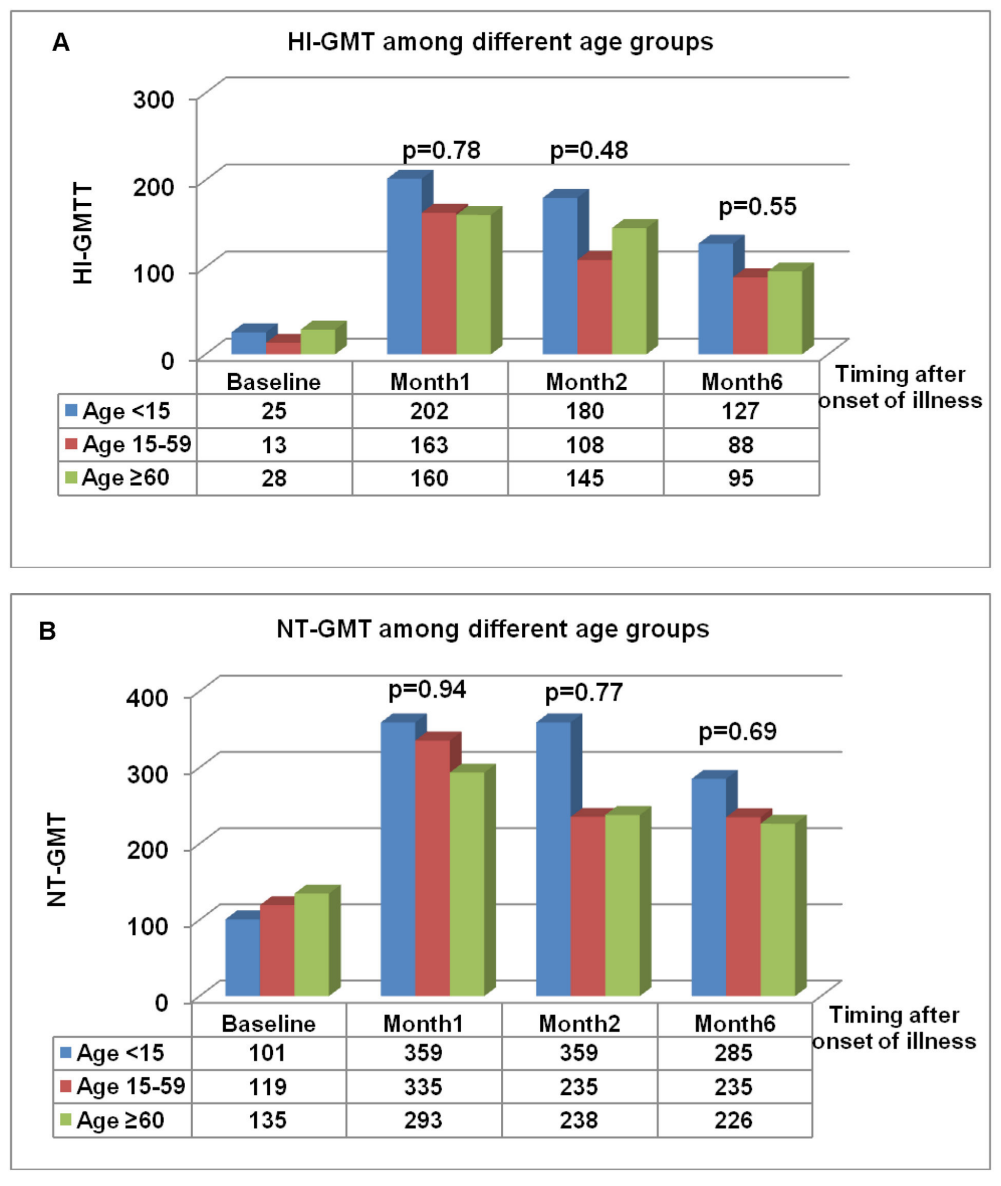
Geometric mean titers (GMT) of antibodies among different age groups. A) GMT of hemagglutination inhibition antibodies (HI-GMT) and B) GMT of neutralization antibodies (NT-GMT) among three age groups (Age < 15, 15-59 and ≥ 60) of 59 patients infected with influenza A (H1N1) pdm09 virus.

Difference of HI-GMT and NT-GMT for those <15, 15-59 and 60 are shown in Figure 3A and 3B, respectively. GMT of HI and NT in those younger than 15 tends to be higher than those in other age groups. However, there was no significant difference of HI and NT GMT among the three age groups at each time point. 

### Clinical characteristics and antibody response of influenza A (H1N1) pdm09 patients with and without pneumonia

Table 4 compares characteristics and antibody responses of influenza A (H1N1) pdm09 patients with and without pneumonia. With the two-sample K-S test, no differences were found in distributions of data between two groups. There was no significant difference of those with and without pneumonia in age (*p*=0.662), gender ratio (*p*=0.282), underlying disease (*p*=0.793), duration from illness to treatment (*p*=0.315), and duration of antiviral treatment (*p*=0.315). The HI and NT GMT of those with pneumonia increased to the peak at 1 month while those without pneumonia peaked at 2 months after onset of illness. Those with pneumonia had a higher HI-GMT than those without pneumonia at 1 month (264 vs. 117, *p*=0.005), 2 months (212 vs. 159, *p*=0.012), and up to 6 months (160 vs. 82, *p*=0.024). The NT-GMT of those with pneumonia was significantly higher than those without pneumonia only at month 2 after illness (412 vs. 245, *p*=0.022). However, when analyzing only adult patients, we found a significant difference in age between those with and without pneumonia (48 [24,70] vs. 28 [16,68], respectively, *p*=0.015); we therefore adjusted for age as a confounder in a multiple logistic regression considering the effects of antibody titer on presence/absence of pneumonia (Table 5). Gender may also be a confounder in this analysis, even though no significant difference was found between those with and without pneumonia. After further adjustment for age, gender, underlying disease, duration from illness to treatment, and duration of antiviral treatment, we found that the relationship between having and not having influenza A (H1N1) pdm09-associated pneumonia and antibody titer remained the same in all analyses (Table 5). 

**Table 4:** Characteristics and antibody response of influenza A (H1N1) pdm09 patients with and without pneumonia (n=59).

		Patients With Pneumonia (n=25)		Patients Without Pneumonia (n=34)		*p*-value
Age (years)*		38 (5, 70)		28 (16, 68)		0.662
Gender, female, n (%)		14 (56)		24 (70.6)		0.282
Underlying disease, n (%)		14 (56)		17 (50)		0.793
Duration from illness to treatment (days)*		2 (1, 6)		2 (0, 7)		0.315
Duration of antiviral treatment (days)*		5 (4, 10)		5 (4, 5)		0.315

HI assay		No. tested	GMT(95% CI)	No. tested	GMT(95% CI)	*p*-value
	Baseline	25	21 (17, 24)	34	13 (10, 15)	0.163
	1 month	25	264 (166, 361)	33	117 (90, 143)	0.005
	2 month	22	212 (144, 281)	31	159 (137, 181)	0.012
	6 month	24	160 (117, 203)	32	82 (66, 97)	0.024
mNT assay						
	Baseline	25	139 (109, 169)	34	106 (86, 127)	0.132
	1 month	25	499 (113, 885)	33	244 (161, 326)	0.014
	2 month	22	412 (261, 563)	31	245 (188, 302)	0.022
	6 month	24	370 (244, 496)	32	241 (186, 297)	0.072

*Reported as median (rank) Abbreviation: GMT=geometric mean titer; CI=confidence interval; Ab=antibody; NT =neutralization; HI=hemagglutination inhibition

**Table 5:** Multiple logistic regression of potential factors for assessing association between having influenza pneumonia and the difference of antibody response.

		Odds ratio (95% CI )					
		Age adjusted	*p*-value	Age and sex adjusted	*p*-value	Multivariate adjusted	*p*-value^†^
HI assay							
	Baseline	1.008 (0.977, 1.039)	0.319	1.009 (0.997, 1.013)	0.243^‡^	1.006 (0.996, 1.016)	0.217
	1 month	1.004 (1.001, 1.006)	0.011	1.004 (1.001, 1.007)	0.007	1.004 (1.001, 1.007)	0.014
	2 month	1.004 (1.001, 1.007)	0.021	1.004 (1.001, 1.008)	0.013	1.005 (1.001, 1.008)	0.016
	6 month	1.005 (1.001, 1.009)	0.024	1.005 (1.001, 1.010)	0.018^‡^	1.005 (1.001, 1.009)	0.017
mNT assay							
	Baseline	1.001 (0.997, 1.006)	0.509	1.002 (0.997, 1.006)	0.420	1.001 (0.996, 1.006)	0.719
	1 month	1.001 (1.000, 1.002)	0.055	1.001 (1.000, 1.002)	0.059^‡^	1.001 (1.000, 1.002)	0.094
	2 month	1.002 (1.000, 1.004)	0.037	1.002 (1.000, 1.004)	0.039	1.002 (1.000, 1.004)	0.075^‡^
	6 month	1.002 (1.000, 1.004)	0.063	1.002 (1.000, 1.005)	0.047	1.002 (1.000, 1.005)	0.087

^†^ Analyses adjusted for all factors listed in Table 4

^‡^ The data did not fit this model well (Hosmer-Lemeshow test had p value <0.05)

## Discussion

This study characterized serum antibody response against influenza A (H1N1) pdm09 virus induced by natural infection and investigated the difference of antibody response regarding the patients’ age group and severity of the disease. Our study was done between February and October 2010, between the pandemic and post pandemic flu season, which was declared by WHO in August 2010. Although the patients during the post-pandemic season were older and likely to have more chronic disease [16], the demographics of our patients were similar to that of those of patients in the pandemic period. Most patients were between 15 to 60 years of age and only half of them had underlying disease. Four patients in our study received seasonal trivalent inactivated influenza vaccine without the component of influenza A (H1N1) pdm09 strain which would have induced little or no cross reactivity of antibody to the influenza A (H1N1) pdm09 in any age group [17]. Two patients got vaccine that contained influenza A (H1N1) pdm09 strain approximately the same time as the symptoms onset; therefore, the antibody response induced by natural infection should have exceeded those by vaccination [18]. Most of our patients were hospitalized and no fatal cases were observed, which was similar to the epidemiological studies of outbreaks of influenza A (H1N1) pdm09 reported in Asia with only 1.6% case fatality [19].

Our results show that approximately 90% of patients produce protective antibodies after natural infection. This result was consistent with previous studies which reported that only 10% of confirmed cases failed to develop protective levels of HI or NT antibodies [20]. Previous studies by Wang reported a significant drop of antibody positive rate, from 100% to 34% at 6 months [21] while those of Hung found that only 70% of patients can maintain the protective level over 1 to 4 months [14]. In contrast, almost 90% of all patients maintained seroprotection at HI above 1:40 or NT above 1:160 for up to 6 months. This difference may correlate with various degrees of disease severity. The previous studies included patients with mild illness whereas our patients were more severely affected with 83% hospitalization which could resulted in a stronger antibody response. 

Seroconversion was observed in about 81% of our participants by HI and 54% by mNT assay. The study by Chen and colleagues found that the seroconversion rate determined by HI could vary from 59% to 82% depending on the appropriate timing of baseline and convalescent blood samplings [22]. Unlike the study by Chen, we collected baseline samples as early as 7 days after onset of illness. We found that the seroconversion rate is higher in those with low HI titers at baseline which is in contrast to what was observed by Chen but similar to the study by Soonawala [23]. In addition, approximately 30% and 50% of participants had baseline HI and NT titers, respectively at or above the threshold that defines seroprotection, which is fairly high when compared to other studies that varied from 0 to approximately 30% [23]. It must be noted that only 10% of our patients were vaccinated against seasonal influenza prior to the pandemic influenza infection so it seems likely that recent seasonal influenza infection might have accounted for a high baseline antibody level as prior infection with seasonal influenza may induce cross reactive antibody, which may reduce lethality from pandemic influenza infection but are not sufficient to prevent the infection [24].

We also demonstrated that the geometric mean titers (GMT) of HI and NT follow a similar pattern over a 6-month period. The level of HI antibody peaks at 1 month, then slightly drops at 2 months, and declines significantly at 6 months while those of NT antibodies did not decline markedly at 6 months after its peak. Similar results were reported by Chen who reported the highest HI-GMT (123, 95% CI 43-356) at days 30-39 after illness onset [22] and by Wang who found a significant decrease of HI-GMT at 6 months [21]. The NT-GMT in our study is two to three times higher than HI-GMT throughout the 6 month period which is similar to those described by Veguilla and colleagues who predicted that the NT titer was generally two to four fold higher than HI titers [5]. 

Even though a previous study found a lower antibody response in the older age group after natural infection [18], there was no evidence of a difference in antibody response between the patients age 60 or older and the other age groups in our study. Also, no significant difference was found between the GMT of those younger than 15 years and that of other age groups which were similar to a previous study [13]. (17) This may be partly explained by the limited number of participants in the extreme age groups. 

The strength of the immune response from natural infection also seems to be correlated with the severity of the disease. In our study, the antibody titers in those with pneumonia were much higher and reached the peak much faster than those without pneumonia, which was similar to the study by Mak who demonstrated that severe cases showed a higher GMT than mild disease until 3 weeks after infection [25]. Notably, we clearly observed a considerably higher level of HI-GMT in those with pneumonia than those without pneumonia up to 6 months after illness. In addition, previous studies have also identified the difference in morbidity as well as in immune response following vaccination by age and gender [26]. The morbidity rates of 2009 pandemic influenza A H1N1 were higher for males than females at younger and older ages [27]. A higher geometric mean titer responses following seasonal trivalent inactivated vaccination was also observed in women for all age, regardless of dose or influenza strain [12]. After adjusting for age and gender or all other factors, we clearly demonstrated an independent association between having pneumonia and difference of antibody response after naturally acquired A (H1N1) pdm09 infection. 

A previous study demonstrated that convalescent plasma therapy could reduce mortality rate for patients with severe H1N1 2009 infection whose condition was deteriorating despite standard antiviral therapy [28]. Thus, it is important to determine which patients have a high level of neutralizing antibodies as they may be good donors of plasma for treatment of severe pandemic H1N1 influenza for which no good treatment exists.

## Conclusions

The study reported herein indicated that neutralizing antibodies persist for a substantial period after disease recovery. This study clearly shows that the seroprotection rate and GMT of antibodies to influenza A (H1N1) pdm09, particularly in patients with pneumonia, rose rapidly and remained high for up to 6 months. The results from our study could have a practical value for use in determining appropriate donors and timing to obtain convalescent plasma for adjunctive treatment of seriously ill patients with pandemic H1N1 influenza. 

## Supporting Information

Table S1 **Clinical characteristics and antibody response of 59 patients with A (H1N1) pdm09 infection.**
(DOCX)
