# Supplementary material for: Serological Response of Patients with Influenza A (H1N1) pdm09-Associated Pneumonia: An Observational Study
Source: PLoS One. 2013 Nov 27;8(11):e81436. doi: 10.1371/journal.pone.0081436 (PMC3842268; doi:10.1371/journal.pone.0081436)
Supplement: Table S1 — Clinical characteristics and antibody response of 59 patients with A (H1N1) pdm09 infection. (DOCX) [file pone.0081436.s001.docx]

| **Table S1. Clinical characteristics and antibody response of 59 patients with A (H1N1) pdm09 infection** | | | | | | | | | | | | | | | | | | | | | | | | | | |  |
| --- | --- | --- | --- | --- | --- | --- | --- | --- | --- | --- | --- | --- | --- | --- | --- | --- | --- | --- | --- | --- | --- | --- | --- | --- | --- | --- | --- |
| ID | age | sex | underlying | pneumonia | nt_bs | nt_v1 | nt_v2 | nt_v3 | | | hi_bs | | | | | | | hi_v1 | | | | | | hi_v2 | hi_v3 | Dur_illtotreat | Dur_treat |
| 1 | 56 | 1 | 1 | 1 | 80 | 640 | 640 | 640 | | | 20 | | | | | | | 320 | | | | | | 320 | 320 | 1 | 5 |
| 2 | 19 | 1 | 1 | 0 | 80 | 320 | 320 | 160 | | | 20 | | | | | | | 160 | | | | | | 160 | 80 | 2 | 4 |
| 3 | 28 | 2 | 0 | 1 | 160 | 640 | 640 | 640 | | | 20 | | | | | | | 320 | | | | | | 320 | 320 | 6 | 5 |
| 4 | 26 | 2 | 0 | 1 | 160 | 320 | 320 | 160 | | | 160 | | | | | | | 160 | | | | | | 160 | 40 | 1 | 5 |
| 5 | 24 | 2 | 0 | 1 | 80 | 320 | 320 | 160 | | | 5 | | | | | | | 320 | | | | | | 320 | 160 | 2 | 4 |
| 6 | 54 | 2 | 0 | 0 | 160 | 80 | 160 | 80 | | | 5 | | | | | | | 160 | | | | | | 160 | 80 | 3 | 5 |
| 7 | 61 | 2 | 0 | 0 | 80 | 320 | 640 | 640 | | | 5 | | | | | | | 640 | | | | | | 640 | 640 | 2 | 5 |
| 8 | 24 | 1 | 0 | 1 | 40 | 40 | 80 | 80 | | | 5 | | | | | | | 40 | | | | | | 40 | 40 | 2 | 5 |
| 9 | 52 | 2 | 0 | 1 | 160 | 640 | 640 | 640 | | | 80 | | | | | | | 640 | | | | | | 640 | 640 | 1 | 4 |
| 10 | 40 | 2 | 1 | 0 | 80 | 640 | 320 | 320 | | | 5 | | | | | | | 320 | | | | | | 320 | 160 | 1 | 5 |
| 11 | 26 | 2 | 0 | 0 | 80 | 80 | 160 | 160 | | | 10 | | | | | | | 40 | | | | | | 40 | 80 | 2 | 4 |
| 12 | 65 | 1 | 1 | 0 | 160 | 160 |  |  | | | 20 | | | | | | | 80 | | | | | |  |  | 6 | 4 |
| 13 | 26 | 1 | 0 | 0 | 40 | 160 | 160 | 160 | | | 10 | | | | | | | 80 | | | | | | 80 | 80 | 2 | 5 |
| 14 | 28 | 2 | 0 | 0 | 160 | 160 | 160 | 160 | | | 320 | | | | | | | 160 | | | | | | 160 | 80 | 1 | 5 |
| 15 | 20 | 2 | 0 | 0 | 320 | 320 | 160 | 160 | | | 40 | | | | | | | 80 | | | | | | 80 | 80 | 2 | 5 |
| 16 | 48 | 2 | 0 | 1 | 160 | 1,280 | 1,280 | 1,280 | | | 10 | | | | | | | 640 | | | | | | 640 | 320 | 4 | 6 |
| 17 | 28 | 2 | 0 | 0 | 160 | 160 | 80 | 80 | | | 40 | | | | | | | 40 | | | | | | 40 | 40 | 1 | 5 |
| 18 | 26 | 1 | 1 | 0 | 80 | 80 | 80 | 80 | | | 5 | | | | | | | 40 | | | | | | 40 | 40 | 2 | 5 |
| 19 | 28 | 2 | 1 | 0 | 80 | 320 | 640 | 320 | | | 5 | | | | | | | 320 | | | | | | 160 | 160 | 2 | 4 |
| 20 | 62 | 2 | 1 | 0 | 40 | 320 | 320 | 640 | | | 5 | | | | | | | 320 | | | | | | 160 | 160 | 1 | 5 |
| 21 | 28 | 1 | 0 | 0 | 80 | 320 | 320 | 160 | | | 20 | | | | | | | 80 | | | | | | 80 | 40 | 3 | 5 |
| 22 | 19 | 2 | 0 | 0 | 40 | 160 | 160 | 320 | | | 10 | | | | | | | 80 | | | | | | 80 | 80 | 2 | 5 |
| 23 | 38 | 1 | 1 | 1 | 80 | 5,120 |  | 2,560 | | | 5 | | | | | | | 1,280 | | | | | |  | 640 | 2 | 5 |
| 24 | 19 | 1 | 1 | 0 | 80 | 1,280 | 640 | 640 | | | 10 | | | | | | | 320 | | | | | | 320 | 320 | 2 | 5 |
| 25 | 68 | 1 | 1 | 0 | 40 | 160 | 160 | 160 | | | 5 | | | | | | | 20 | | | | | | 20 | 10 | 4 | 5 |
| 26 | 28 | 2 | 0 | 0 | 80 | 160 |  | 160 | | | 20 | | | | | | | 160 | | | | | |  | 160 | 1 | 5 |
| 27 | 19 | 2 | 0 | 0 | 160 | 160 | 160 | 160 | | | 40 | | | | | | | 80 | | | | | | 40 | 40 | 5 | 5 |
| 28 | 54 | 2 | 0 | 1 | 160 | 2,560 | 640 | 320 | | | 40 | | | | | | | 640 | | | | | | 640 | 320 | 5 | 5 |
| 29 | 27 | 2 | 0 | 0 | 80 | 640 | 640 | 320 | | | 40 | | | | | | | 320 | | | | | | 320 | 320 | 1 | 5 |
| 30 | 19 | 2 | 0 | 0 | 40 | 160 | 160 | 320 | | | 40 | | | | | | | 160 | | | | | | 160 | 160 | 1 | 5 |
| 31 | 60 | 2 | 1 | 1 | 80 | 80 | 160 | 160 | | | 5 | | | | | | | 160 | | | | | | 160 | 160 | 2 | 4 |
| 32 | 28 | 2 | 1 | 0 | 40 | 80 | 80 | 80 | | | 5 | | | | | | | 10 | | | | | | 10 | 10 | 3 | 4 |
| 33 | 47 | 2 | 1 | 1 | 160 | 1,280 | 640 | 640 | | | 40 | | | | | | | 640 | | | | | | 640 | 320 | 0 | 5 |
| 34 | 27 | 2 | 1 | 0 | 40 | 160 | 320 | 320 | | | 10 | | | | | | | 320 | | | | | | 160 | 160 | 2 | 5 |
| 35 | 27 | 2 | 1 | 0 | 160 | 1,280 | 640 | 320 | | | 10 | | | | | | | 320 | | | | | | 160 | 160 | 1 | 4 |
| 36 | 35 | 2 | 1 | 0 | 160 | 320 | 320 |  | | | 40 | | | | | | | 640 | | | | | | 320 |  | 2 | 5 |
| 37 | 60 | 1 | 1 | 0 | 40 | 320 |  | 160 | | | 5 | | | | | | | 10 | | | | | |  | 5 | 3 | 5 |
| 38 | 48 | 1 | 0 | 1 | 640 | 5,120 | 1,280 | 320 | | | 20 | | | | | | | 320 | | | | | | 320 | 40 | 5 | 9 |
| 39 | 70 | 1 | 1 | 1 | 320 | 320 | 320 | 320 | | | 5 | | | | | | | 160 | | | | | | 160 | 80 | 0 | 5 |
| 40 | 49 | 1 | 1 | 1 | 160 | 160 | 320 | 320 | | | 10 | | | | | | | 80 | | | | | | 80 | 80 | 5 | 4 |
| 41 | 29 | 2 | 0 | 0 | 160 | 160 | 160 | 320 | | | 10 | | | | | | | 80 | | | | | | 80 | 80 | 1 | 5 |
| 42 | 16 | 2 | 1 | 0 | 320 | 160 | 160 | 640 | | | 5 | | | | | | | 40 | | | | | | 40 | 40 | 2 | 5 |
| 43 | 65 | 2 | 0 | 1 | 320 | 2,560 | 1,280 | 640 | | | 5 | | | | | | | 640 | | | | | | 640 | 320 | 7 | 5 |
| 44 | 59 | 2 | 0 | 0 | 320 | 320 | 320 | 320 | | | 5 | | | | | | | 160 | | | | | | 80 | 80 | 2 | 5 |
| 45 | 29 | 1 | 1 | 0 | 80 | 80 | 80 | 80 | | | 5 | | | | | | | 80 | | | | | | 80 | 40 | 1 | 4 |
| 46 | 34 | 2 | 0 | 1 | 160 | 1,280 |  | 640 | | | 40 | | | | | | | 640 | | | | | |  | 320 | 5 | 5 |
| 47 | 22 | 2 | 1 | 0 | 320 | 320 | 320 | 640 | | | 10 | | | | | | | 160 | | | | | | 80 | 80 | 2 | 5 |
| 48 | 36 | 2 | 1 | 0 | 320 | 320 | 320 | 320 | | | 5 | | | | | | | 80 | | | | | | 80 | 80 | 1 | 5 |
| 49 | 37 | 1 | 0 | 0 | 160 | 2,560 | 1,280 | 640 | | | 10 | | | | | | | 640 | | | | | | 640 | 320 | 2 | 5 |
| 50 | 43 | 2 | 1 | 1 | 80 | 80 | 160 | 80 | | | 10 | | | | | | | 80 | | | | | | 80 | 40 | 1 | 5 |
| 51 | 38 | 2 | 0 | 0 | 640 |  | 320 | 640 | | | 160 | | | | | | |  | | | | | | 160 | 160 | 3 | 5 |
| 52 | 30 | 2 | 1 | 1 | 80 | 160 | 160 | 160 | | | 40 | | | | | | | 80 | | | | | | 20 | 20 | 0 | 5 |
| 53 | 55 | 2 | 1 | 1 | 320 | 640 | 1,280 | 1,280 | | | 640 | | | | | | | 320 | | | | | | 640 | 640 | 1 | 6 |
| 54 | 5 | 1 | 1 | 1 | 160 | 640 |  |  | | | 40 | | | | | | | 320 | | | | | |  |  | 4 | 5 |
| 55 | 6 | 1 | 1 | 1 | 80 | 320 | 320 | 320 | | | 5 | | | | | | | 320 | | | | | | 160 | 160 | 5 | 5 |
| 56 | 12 | 2 | 0 | 1 | 160 | 640 | 640 | 640 | | | 80 | | | | | | | 320 | | | | | | 320 | 320 | 4 | 10 |
| 57 | 13 | 1 | 1 | 1 | 160 | 160 | 160 | 160 | | | 80 | | | | | | | 40 | | | | | | 40 | 40 | 3 | 4 |
| 58 | 13 | 1 | 1 | 1 | 160 | 160 | 160 | 160 | | | 5 | | | | | | | 160 | | | | | | 160 | 160 | 3 | 4 |
| 59 | 6 | 1 | 1 | 1 | 80 | 640 | 640 | 640 | | | 40 | | | | | | | 640 | | | | | | 320 | 320 | 1 | 7 |
|  |  |  |  |  |  |  |  |  | | |  | | | | | | |  | | | | | |  |  |  |  |
| This is a data set for serological response of patients with influenza A (H1N1) pdm09-associated pneumonia | | | | | | | | | | | | | | | | | | | | |  |  |  |  |  |  |  |
| : An observational study | | | | | | | | | |  | |  |  |  | |  | | |  | |  |  |  |  |  |  |  |
| The data is provided by Nasikarn Angkasekwinai: nasikarn@gmail.com | | | | | | | | | | | | | |  | |  | | |  | |  |  |  |  |  |  |  |
| Please contact Nasikarn if you plan to conduct analyses on these data for publication | | | | | | | | | | | | | | | | | | |  | |  |  |  |  |  |  |  |
|  | | |  | | | | | | |  | |  |  | |  | |  | | |  | |  |  |  |  |  |  |
| **Variable name** | | | **Variable description** | | | | | | | | |  |  |  | |  | | |  | |  |  |  |  |  |  |  |
| ID | | | Patient ID code | | | | | | | | |  |  |  | |  | | |  | |  |  |  |  |  |  |  |
| age | | | Age | | | | | |  |  | |  |  |  | |  | | |  | |  |  |  |  |  |  |  |
| sex | | | Sex: 1 is male | | | | | | | | |  |  |  | |  | | |  | |  |  |  |  |  |  |  |
| underlying | | | Underlying disease | | | | | | | | |  |  |  | |  | | |  | |  |  |  |  |  |  |  |
| pneumonia | | | Pneumonia | | | | | | |  | |  |  |  | |  | | |  | |  |  |  |  |  |  |  |
| nt_bs | | | mNT at baseline | | | | | | | | |  |  |  | |  | | |  | |  |  |  |  |  |  |  |
| nt_v1 | | | mNT at 1 month after onset of illness | | | | | | | | | |  |  | |  | | |  | |  |  |  |  |  |  |  |
| nt_v2 | | | mNT at 2 month after onset of illness | | | | | | | | | |  |  | |  | | |  | |  |  |  |  |  |  |  |
| nt_v3 | | | mNT at 6 month after onset of illness | | | | | | | | | |  |  | |  | | |  | |  |  |  |  |  |  |  |
| hi_bs | | | HI at baseline | | | | | | |  | |  |  |  | |  | | |  | |  |  |  |  |  |  |  |
| hi_v1 | | | HI at 1 month after onset of illness | | | | | | | | | |  |  | |  | | |  | |  |  |  |  |  |  |  |
| hi_v2 | | | HI at 2 month after onset of illness | | | | | | | | | |  |  | |  | | |  | |  |  |  |  |  |  |  |
| hi_v3 | | | HI at 6 month after onset of illness | | | | | | | | | |  |  | |  | | |  | |  |  |  |  |  |  |  |
| Dur_illtotreat | | | Duration from onset of illness to treatment | | | | | | | | | |  |  | |  | | |  | |  |  |  |  |  |  |  |
| Dur_treat | | | Duration of treatment | | | | | | | | |  |  |  | |  | | |  | |  |  |  |  |  |  |  |
|  | | |  | | | | | |  |  | |  |  |  | |  | | |  | |  |  |  |  |  |  |  |
|  | | |  | | | | | |  |  | |  |  |  | |  | | |  | |  |  |  |  |  |  |  |
